# Effectiveness of Moral Developmental Interventions for Youth Engaged in Delinquent Behavior: A Meta-Analysis

**DOI:** 10.1177/0306624X231172648

**Published:** 2023-05-22

**Authors:** Evelyn Heynen, Larissa Hoogsteder, Eveline van Vugt, Frans Schalkwijk, Geert-Jan Stams, Mark Assink

**Affiliations:** 1 Open University, Heerlen, The Netherlands; 2 University of Amsterdam, The Netherlands

**Keywords:** moral development, behavioral interventions, conscience, meta-analysis

## Abstract

There is vast empirical evidence showing that juvenile delinquency is associated with delays in moral development, including moral judgment, empathy, and self-conscious emotions (guilt and shame). Consequently, interventions have been developed that target moral development of juvenile delinquents to reduce criminal offense recidivism. However, a comprehensive synthesis of studies examining the effectiveness of these interventions was not yet available. The present meta-analysis of (quasi-)experimental research therefore examined the effects of interventions that target moral development of youth engaged in delinquent behavior. Interventions that targeted moral judgment (11 studies and 17 effect sizes) showed a significant and small-to-medium effect on moral judgment (*d* = 0.39), with intervention type as a significant moderator, but no significant effect on recidivism (*d* = 0.03; 11 studies and 40 effect sizes). No (quasi-)experimental studies were found that targeted guilt and shame in juvenile offenders, and an insufficient number of studies (i.e., only two) were found to conduct a meta-analysis of interventions that target empathy. The discussion focuses on potential ways to improve moral development interventions for youth engaged in delinquent behavior, and provides suggestions for future research.

## Introduction

In line with Bauman (1993) and Beauchamp and Childress (2019), morality can be defined as the aspect of human thought, feeling, and action that pertains to doing right (fairness and justice) and good (benevolence and caring). Aspects of moral development are moral judgment (referring to the insights one has into moral rules and the well-being of others), empathy (referring to the ability to understand another’s emotions and feelings, or share another’s emotional state), and the self-conscious emotions of guilt and shame (Schalkwijk et al., 2016).

Several meta-analyses revealed that youth engaged in delinquent behavior display a delay in their moral development, which is most prominent in their levels of moral judgment (Gibbs et al., 2007; Stams et al., 2006), and less prominent in their levels of empathy (Van Langen et al., 2014) and self-conscious emotions (Spruit et al., 2016). In fact, it has been found that particularly moral cognition, including moral judgment and cognitive empathy, is more strongly associated with criminal offense recidivism than moral (self-conscious) emotions, such as affective empathy, shame, and guilt (Spruit et al., 2016; Van Vugt et al., 2011). These findings suggest that interventions targeting - among other things - moral development may be useful in the treatment of youth engaged in delinquent behavior.

Interventions that target moral development can be understood as forms of treatment that are specifically aimed at positively influencing the moral development of juvenile offenders to reduce recidivism. This means, for example, that “restorative justice” interventions, aiming to repair the moral breach between the offender and society, are excluded from the current overview. The restorative justice approach (e.g., victim-offender mediation and sentencing cycles) does not specifically aim to reduce criminal recidivism by stimulating moral development of (juvenile) offenders.

Throughout the years, different types of interventions that target juvenile offenders’ moral development have emerged. A brief overview is provided here. *EQUIP* is a group intervention that aims to reduce recidivism by improving social skills, increasing moral judgment, and reducing cognitive distortions (Gibbs et al., 1995; Potter et al., in press). Studies into the effects of EQUIP on detained adolescents showed positive effects on all relevant outcomes in the USA, but not in Europe (Van Stam et al., 2014).

*Aggression Replacement Training* (ART; Goldstein et al., 1987) is a group intervention comprising three components; social skills training, aggression regulation training, and group discussions about moral dilemmas to increase the level of moral judgment. The training can be embedded in more comprehensive behavioral interventions, such as EQUIP. The evidence for the effectiveness of ART in stimulating moral development in terms of moral judgment and reducing aggression and recidivism is unconvincing to date. Study designs are generally weak and/or ART is investigated by non-independent researchers who, as program authors might be less objective, as shown by two reviews, with 16 and 10 studies, respectively (Brännström et al., 2016; Ensafdaran et al., 2019). ART and EQUIP can both be considered as multi-component programs.

*Reasoning and Rehabilitation* (R&R; Goldstein et al., 1998; Ross et al., 1988) is a form of cognitive behavioral therapy that aims to improve social skills, change criminogenic thinking patterns, and improve moral reasoning. To date, research has only studied the effects of R&R on recidivism, for which a small positive effect was found, without focusing on intermediary outcomes, such as moral judgment and empathy (Tong & Farrington, 2006). Furthermore, many studies on R&R have a weak study design, which means that many alternative explanations for the positive effects of the intervention cannot be ruled out, or they were conducted by non-independent researchers who could potentially benefit from positive outcomes of R&R.

Finally, *Moral Reconation Therapy* (MRT: Little & Robinson, 1988) is a group intervention that aims to re-educate clients socially, morally, and behaviorally, and to instill appropriate goals, motivation, and values. The results of a meta-analysis of which only two studies relate to youth engaged in delinquent behavior show a small positive overall effect of MRT on recidivism. The effect was moderated by age of the juveniles, residential versus ambulant contexts, type of study design, length of follow-up time, and whether or not researchers were involved with the development of MRT (Ferguson & Wormits, 2012). This study did not provide information on the effects of MRT on moral judgment. A comprehensive review of studies examining the effectiveness of moral development interventions for youth engaged in delinquent behavior is not yet available. Outcomes of studies investigating the effects of interventions targeting moral development of youth engaged in delinquent behavior yielded equivocal results. It is likely that differences in results across studies can be explained by moderating factors, such as study design, type of offense (violent or non-violent), participant characteristics (gender, age, and ethnicity), type of intervention, the instruments used to assess moral development, and publication characteristics. For example, previous research has found a moderate and significant inverse relation between quality of the research design and effectiveness of judicial interventions in crime and justice (Weisburd et al., 2001; Welsh et al., 2011). Stams et al. (2006) showed that the type of measurement moderates the association between moral judgment and delinquency, in the sense that production measures (semi-structured interviews assessing self-produced moral argumentations) yielded stronger associations than recognition measures (multiple-choice questionnaires assessing the level of moral judgment). In addition, study outcomes have been shown to depend on the assessment of delinquency, with differences between self-report and official registrations of delinquent behavior (Asscher et al., 2014; Breuk et al., 2007).

It is plausible to assume that when the moral development of young first-offenders, and offenders who commit minor and non-violent offenses is targeted, larger effects are found than when the moral development of youth showing more severe, violent, or persistent criminal behavior is addressed, because in the latter case, delinquency is caused and maintained by multiple risk factors in different risk domains rather than factors that pertain to moral development alone (Andrews & Bonta, 2006; Assink et al., 2015). Moreover, deficiencies in moral development may be a far less important criminogenic factor in youth engaged in severe and persistent delinquent behavior than externalizing-inducing traumatic experiences (Hoogsteder et al., 2022), such as child maltreatment (Asscher et al., 2014), or disruptive behavioral disorders, particularly conduct disorder (Wibbelink et al., 2017). Especially poor inhibition and lack of impulse control have been related to disorders that have a relatively high prevalence in youth engaged in delinquent behavior, such as ADHD, autism spectrum disorder, and conduct disorder (Puiu et al., 2018). However, a drawback of interventions that target inhibition and impulse control outside a moral context may be that the intervention program will just succeed in producing more effective criminals. This may be one of the reasons why social skills interventions for youth engaged in delinquent behavior produce only small positive changes (Van der Stouwe et al., 2021). Finally, it is important to realize that even if juveniles who show delinquent behavior in combination with complex problems are aware of the difference between “right” and “wrong,” they may still opt to engage in (antisocial) behaviors by believing that their behavior serves a higher purpose (justification) or by not taking (full) responsibility for it and placing the blame entirely on another person (Bandura, 2016; Caprara et al., 2014). Blaming the other, “externalizing,” can be seen as a dysfunctional cognition that is frequently used by adolescents with antisocial behaviors.

The objective of the present meta-analysis was therefore to provide more insight in the effects of interventions that aim to improve moral cognition (i.e., moral judgment) and/or moral emotions (i.e., empathy, shame, and guilt) in youth engaged in delinquent behavior to reduce criminal offense recidivism.

## Methods

In conducting and reporting the current review, the 2020 Preferred Reporting Items for Systematic reviews and Meta-Analyses (PRISMA) guidelines were followed (Page et al., 2021). The protocol for study screening and selection was registered in PROSPERO (registration number: 384661).

### Sample of Studies

Studies were included based on three criteria: (1) Studies had to employ an experimental or quasi-experimental design in which an experimental group was compared to a control group. (2) Studies had to focus on youth engaged in delinquent behavior (not young people at risk for offending or deviant behavior), male or female aged from 12 to 23 years, and (3) studies had to evaluate an intervention that aims to improve moral judgment, empathy, or moral self-conscious emotions (i.e., shame or guilt).

### Information Sources and Search Strategy

**Figure 1.**
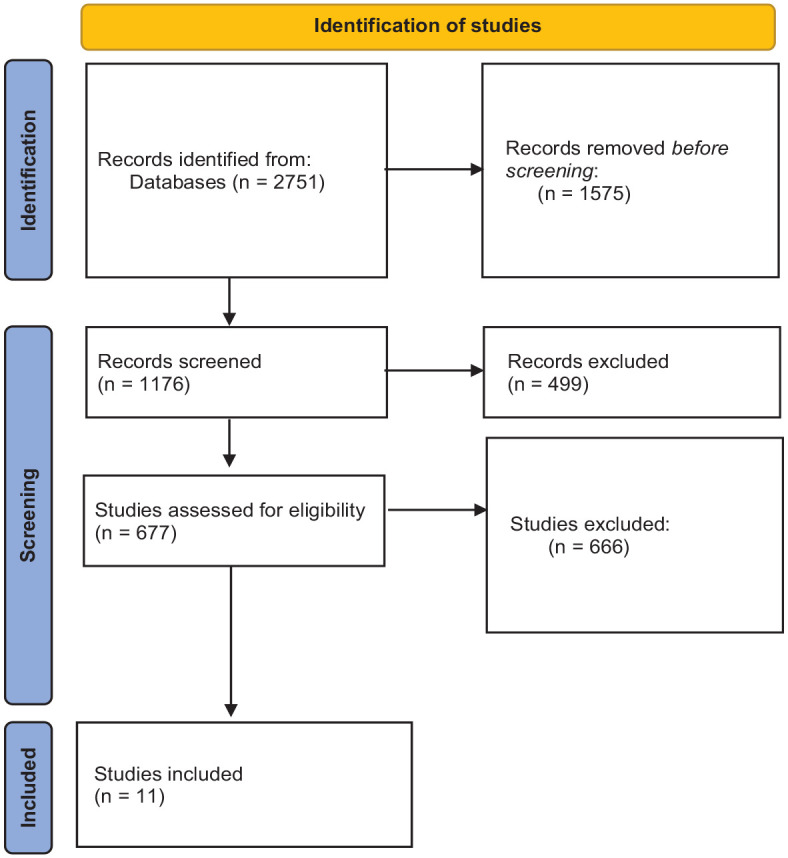
Flowchart.

A comprehensive search of the literature published until February 2022 was conducted to identify research on the effectiveness of interventions that aim to improve moral behavior of youth engaged in delinquent behavior. This search was not restricted in terms of the publication year of primary studies. Studies were identified in three consecutive steps. The first step was to identify studies through keyword searches in the electronic databases Ovid MEDLINE(R) ALL, ERIC, and PsycINFO. The following search string was used: “empath*” or “moral judgment” or “moral reasoning” or guilt or shame or “cognitive distortion” AND (treatment* or intervention* or therapy or psychotherapy) AND (EQUIP or “moral reconation therapy” or “reasoning and rehabilitation” or “aggression replacement training”) AND (delinq* or criminal* or offens* or offenc* or offender* or recidivism) AND (youth* or child* or juvenile* or adolesc* or “young adult”). This search yielded 1,176 studies. After removing the duplicates and limiting the search to journal articles, 677 studies remained. All 677 titles, abstracts, and full texts were screened after which studies not meeting the inclusion criteria were excluded (see Figure 1).

Second, studies were found using the snowball method. This entailed manually screening the reference sections of relevant (already retrieved) articles, relevant narrative reviews, and book chapters. We used Google Scholar to check whether we missed any recently published study that would be eligible for inclusion, but was not identified in the electronic database search. All identified studies that met the inclusion criteria up to February 2022 and could be retrieved were included. Studies were independently screened by two authors. In the end, our search strategy resulted in *k* = 20 studies, from which 57 effect sizes could be extracted. Eleven studies examined the intervention effects on moral judgment (17 ES) and eleven studies examined the intervention effects on criminal recidivism (40 ES). Two of these studies examined both moral judgement and criminal recidivism. Sample sizes for studies on moral judgment ranged from 17 (Haines, 1986; Niles, 1986) to 115 (Helmond et al., 2012), with a mean of 51 participants per dataset (see Appendix 1). For studies on criminal recidivism, the sample sizes ranged from 28 (Moody, 1997) to 738 (Cann et al., 2005), with a mean of 181 participants per dataset (see Appendix 2). The included studies are marked with an asterisk in the Reference list.

#### Study Selection

The articles that remained after deduplication were independently screened by two authors for eligibility on the basis of titles and abstracts. Next, the full-text of the remaining articles were also independently screened by two researchers. Discrepancies were resolved by the involvement of the other authors until consensus was reached. Additional records were retrieved through forward and backward citation searching. All steps were guided by the screening and selection protocol (PROSPERO registration number: 384661). A coding scheme was developed for coding all study design, sample, outcome, and intervention characteristics. In developing this scheme, the guidelines of Lipsey and Wilson (2001) were followed. Coding was done in SPSS (version 28, IBM). Variables were coded that could have a moderating effect on moral reasoning/moral judgment. Different types of moral interventions were evaluated. Regarding the *Intervention Type* we coded ART/EQUIP, Moral discussion group or Other. ART/EQUIP refers to all interventions that are based on ART/EQUIP or are EQUIP oriented. Regarding *study characteristics* we coded study design (RCT or quasi-experimental) and type of control group (TAU, placebo, or no treatment).

Regarding *publication characteristics*, we coded the country in which a primary study was conducted (Europe, USA, or Australia), year of publication, and the impact factor of the journal. We also coded type of measure (production versus recognition measure), in which production measures refer to semi-structured interviews asking participants to produce their own answers to a number of questions, whereas recognition measures refer to questionnaires with items that are answered on a Likert-type scale. As for *participant characteristics*, we coded type of offense (general, minor, violent, sexual, drug related, or status), percentage of White participants (race), gender distribution (only male, only female, or mixed) and mean age of the sample. In coding the offense type, minor offenses refer to stealing or parole violations, whereas violent offenses refer to all types of relatively major offenses, including violence. All included studies used official registrations of delinquency/offenses. Studies not specifying the type of offense were coded as assessing general offenses.

### Calculation of Effect Sizes

Cohen’s *d* was chosen as the common effect size metric. All effects of moral interventions as reported in each primary study were extracted directly or transformed into Cohen’s *d* using the reported statistical information and formulas of Lipsey and Wilson (2001). In most cases, Cohen’s *d* was calculated based on proportions, or means and standard deviations. When raw outcome data were not reported in a study, we transformed a test statistic (*F* value, *z* value, χ^2^ value, or *t* value) into Cohen’s *d*. To control for pre-test differences between an intervention and control group, we calculated effect sizes for both pre-test and post-test outcomes if available, after which pre-test effect sizes were subtracted from post-test effect sizes.

### Statistical Analysis

Two three level meta-analysis were conducted in R (version 3.3, R Core Team, 2021) using a three-level random effects model. In this three-level approach to meta-analysis, dependency of effect sizes is modeled by partitioning the variance in effect sizes into three sources of variance: sampling variance of the observed effect size (Level 1), the variance between effect sizes within studies (Level 2), and the variance between studies (Level 3; Cheung, 2014; Houben et al., 2015; López-López et al., 2014). This three-level approach to meta-analysis permits extraction of all relevant effect sizes from individual studies, so that all information is preserved, and maximum statistical power is achieved in the statistical analysis (Assink et al., 2015). Two separate three-level intercept-only models were built to obtain an overall estimate of the effect of moral interventions on moral judgment and an effect of moral interventions on criminal recidivism in youth engaged in delinquent behavior. To determine whether it was necessary to conduct moderator analyses, two likelihood-ratio-tests were performed to determine the significance of the within- and between-study variance in effect sizes for each meta-analysis. In case of significant variance within and/or between studies, moderator analyses were conducted. Prior to the analyses, continuous variables were centered around their mean, and categorical variables were dummy-coded.

### Publication Bias

Publication bias was examined by testing for asymmetry of a “funnel plot,” which is a graphical representation of the effect size distribution with effect sizes on the horizontal axis plotted against a measure of sample size on the vertical axis (the standard error in the current review). In the absence of bias, the effect sizes form a symmetrical funnel around the overall estimated effect. In case bias is present, asymmetries in the funnel shape can be detected. Missing effect sizes on the right side of the plot indicate selection bias, whereas missing effect sizes on the left side indicate publication bias. To examine funnel plot asymmetry, the trim and fill procedure (Duval & Tweedie, 2000a, 2000b) was performed, which estimates potentially “missing” effect sizes based on the observed effect sizes.

## Findings

No studies were found that examined interventions targeting self-conscious emotions (shame and guilt) with the aim to reduce recidivism in juvenile offenders. Only two studies were found that focused on interventions addressing empathy (Strom et al., 2017; Theriot, 2006), of which Theriot (2006) reported a medium (positive) effect on empathy and varying weak (positive and negative) effects on recidivism.

**Figure 2.**
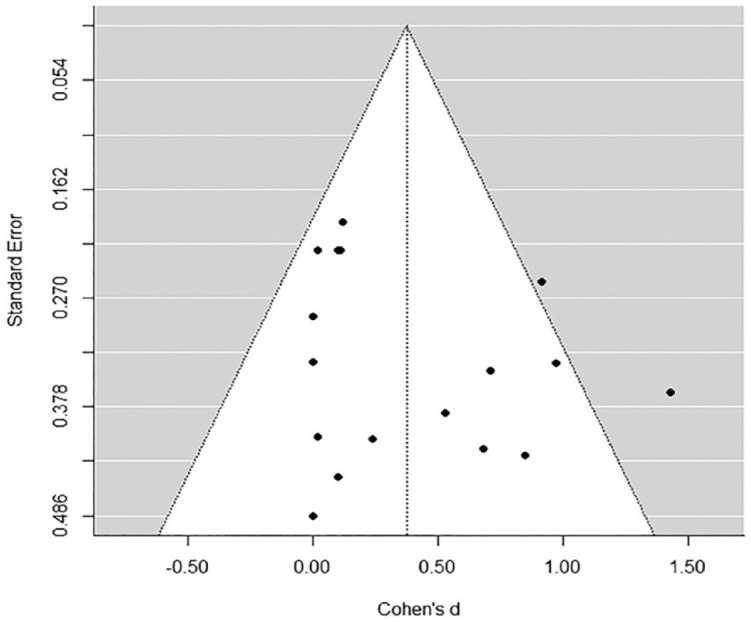
Funnel plot of the moral judgment meta-analysis.

The overall effect of moral development interventions on moral judgment was small-to-medium and significant (*d* = 0.39, *p* = .01; see Table 1 and Appendix 3 for the three-level forest plot of the mean study effect sizes). A trim and fill procedure did not show indications of publication or selection bias (see Figure 2). There was no significant within-study-variance (χ^2^ (1) = 0.000, *p* = 1.00), but there was significant between-study-variance (χ^2^ (1) = 4.16, *p* = .05). Of the total variance, 45.41% was sampling variance (Level 1), 0.00% was within-study variance (Level 2), and 54.59% was between-study-variance (Level 3; see Table 1).

**Table 1.** Overall Intervention Effect on Moral Judgment.

Outcome	*k*	*#*ES	Mean *d*	[95% CI]	*p*-Value	σ^2^_level 2_	σ^2^_level 3_	% var. level 1	% var. level 2	% var. level 3
Moral judgment	11	17	0.39	[0.10, 0.67]	.01	0.000	0.111^*^	45.41	0.00	54.59

*Note. k* = number of studies; *#*ES = number of effect sizes; mean *d* = mean effect size (*d*); CI = confidence interval; σ^2^_level 2_ < variance between effect sizes extracted from the same study; σ^2^_level 3_ < variance between studies; % Var = percentage of variance distributed.

* *p* < .05 (two-tailed).

In an attempt to explain the significant study variance, moderator analyses were conducted (see Table 2). Only “intervention type” was a significant moderator in the sense that the ART/EQUIP mean effect (*d* = 0.08) was significantly lower than the mean effect of other interventions (*d* = 0.58). The results also revealed that the ART/EQUIP mean effect was non-significant, whereas the mean effect of other interventions significantly deviated from zero and was medium in size. Given the modest number of studies and effect sizes that could be synthesized, there was limited statistical power in the omnibus *F* tests. It is therefore relevant to inspect the estimated mean effects rather than only paying attention to the significance of the moderator analyses. First, the estimated mean effect for production measures (*d* = 0.47, *p* < .05) significantly deviated from zero, whereas the effect for recognition measures was non-significant (*d* = 0.32, *ns.*). Second, significant, medium, and positive effects were found in quasi-experimental studies (*d* = 0.49), whereas RCTs produced a mean effect size close to zero (*d* = 0.03), which was not significant. Finally, (borderline) significant and medium effects were found for juveniles committing minor (*d* = 0.50) or general (*d* = 0.42) offenses, but not for juveniles committing violent offenses (*d* = 0.08).

**Table 2.** Moderator Analyses Results in the Meta-Analysis on Moral Judgment.

Variable	*k*	#ES	*B*_0_/*d*	*t*_0_	*B*_1_	*t*_1_	*F* (*df*_1_, *df*_2_)
Intervention							*F* (1, 15) = 5.986*
ART/Equip (RC)	3	5	0.08	0.469			
Other programs	8	12	0.58	4.428***	0.51	2.446*	
Outcome measurement							
Type of measure (RC)							*F* (1, 15) = 0.259
Recognition	7	10	0.32	1.730			
Production	4	7	0.47	2.178*	0.15	0.509	
Study characteristics							
Design							*F* (1, 15) = 2.292
RCT (RC)	3	3	0.03	0.091			
Quasi experimental	8	14	0.49	3.370**	0.47	1.514	
Type of control group							*F*(2, 14) = 0.334
TAU (RC)	3	5	0.30	1.401			
Placebo	2	2	0.59	1.762^+^	0.29	0.742	
No treatment	7	10	0.40	1.925^+^	0.10	0.337	
Participant characteristics							
Percentage White	6	11	0.18	1.740	−0.01	−0.741	*F* (1, 9) = 0.549
Gender							*F* (2, 14) = 0.109
Male (RC)	9	11	0.42	2.576*			
Female	1	2	0.27	0.701	−0.15	−0.386	
Mixed	2	4	0.31	1.025	−0.10	−0.292	
Age	11	17	0.39	2.794*	−0.02	−0.181	*F* (1, 15) = 0.033
Type of offense							*F* (2, 14) = 0.511
General	6	11	0.42	2.308*			
Violent	2	2	0.08	0.243	−0.34	−0.887	
Minor	3	4	0.50	1.810^+^	0.08	0.235	
Publication characteristics							
Country							*F* (2, 14) = 0.227
USA (RC)	7	11	0.37	1.951^+^			
Europe	3	5	0.33	1.361	−0.04	−0.120	
Australia	1	1	0.71	1.392	0.34	0.622	
Year of publication	11	17	0.39	2.747*	0.01	0.528	*F* (1, 15) = 0.279
Impact factor of the journal	11	17	0.39	2.763*	−0.02	−0.223	*F* (1, 15) = 0.053

*Note. k* = number of independent studies; #ES = number of effect sizes; *B*_0_/Cohen’s *d* = intercept; *t*_0_ = *t* value for testing the significance of the mean Cohen’s *d; B*_1_ = estimated regression coefficient (i.e., difference in Cohen’s *d* with reference category); *t*_1_ = test of regression coefficient; *F*(*df*_1_, *df*_2_) = omnibus *F* test; (RC) = reference category.

+ *p* < .10. *p < .05. **p < .01. ***p < .001.

**Figure 3.**
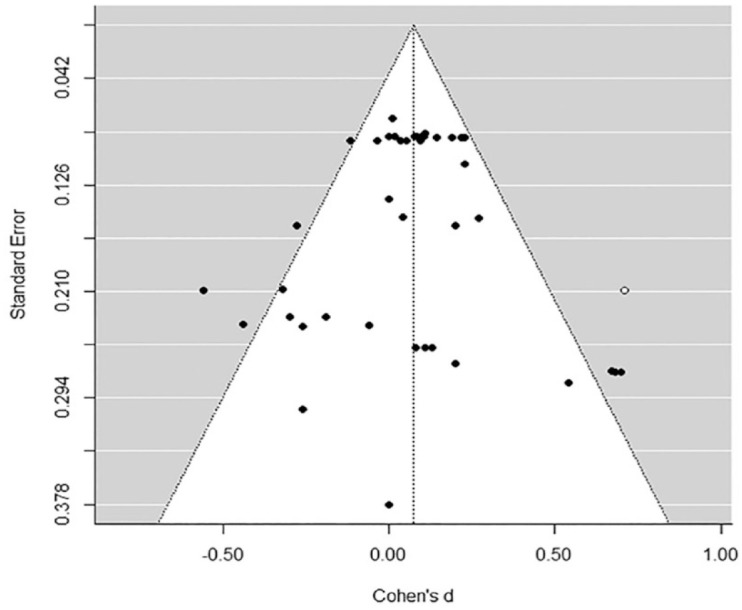
Funnel plot of the criminal recidivism meta-analysis.

The recidivism meta-analysis produced an overall effect of moral interventions on criminal recidivism that was small and not significant (*d* = 0.03, *p* = .71; see Table 3, and Appendix 4, for the three-level forest plot of the mean study effect sizes). A trim and fill procedure did not show indications of publication bias, but only some indication of selection bias given that one effect size from one study was added by the algorithm to the right side of the plot (see Figure 3). After imputing this “missing” effect size to the dataset, an “adjusted” overall effect was estimated which was somewhat larger, but still not significant (*d* = 0.08, *p* = .39; Δ*d* = 0.05). There was no significant within-study-variance (χ^2^ (1) = 0.122, *p* = .73), but there was significant variance in effect sizes between studies (χ^2^ (1) = 12.180, *p* < .001). Of the total variance, 20.06% was sampling variance (Level 1), 1.43% was within-study-variance (Level 2), and 87.51% was between-study variance (Level 3; see Table 3). Moderator analyses did not produce significant effects (See Table 4).

**Table 3.** Overall Intervention Effect on Criminal Recidivism.

Outcome	*k*	*#*ES	Mean *d*	[95% CI]	*p*	σ^2^_level 2_	σ^2^_level 3_	% var. level 1	% var. level 2	% var. level 3
Recidivism	11	40	0.03	[−0.14; 0.20]	.71	0.001	0.057***	20.06	1.43	87.51

*Note. k* = number of studies; #ES = number of effect sizes; mean *d* = mean effect size (Cohen’s *d*); CI = confidence interval; σ^2^_level 2_ = variance between effect sizes extracted from the same study; σ^2^_level 3_ = variance between studies; % Var = percentage of variance distributed.

*** *p* < .001 (one-tailed).

**Table 4.** Moderator Analyses Results in the Meta-Analysis on Criminal Recidivism.

Moderator variable	*k*	#ES	*B*_0_/*d*	*t*_0_^2^	*B*_1_	*t*_1_	*F* (*df*_1_, *df*_2_)
Type of outcome							*F* (1, 38) = 0.175
Re-offense (RC)	11	36	0.04	0.424			
Time to re-offense	4	4	0.00	0.015	−0.04	−0.420	
Type of intervention							*F* (2, 37) = 0.048
Other (RC)	4	22	0.05	0.291			
ART/EQUIP	5	13	0.00	0.014	−0.05	−0.205	
R&R	2	5	0.08	0.364	0.03	0.116	
Study characteristics							
Design							*F* (1, 38) = 0.606
RCT (RC)	2	2	0.20	0.860			
Quasi experimental	9	38	0.00	0.039	−0.20	−0.778	
Type of control group							*F* (1, 38) = 0.129
TAU (RC)	6	14	0.00	0.013			
No treatment	5	26	0.06	0.513	0.06	0.359	
Intention to treat							*F* (1, 38) = 1.761
Yes (RC)	3	20	−0.04	−0.371			
No	8	20	0.06	0.677	0.10	1.327	
Participants characteristics							
Percentage White	8	32	−0.03	−0.230	0.00	0.113	*F* (1, 30) = 0.013
Gender							*F* (1, 34) = 1.311
Male (RC)	6	10	0.06	0.580			
Mixed	4	26	−0.10	−1.040	−0.16	−1.145	
Age	11	40	0.03	0.284	0.00	0.072	*F* (1, 38) = 0.005
Publication characteristics							
Continent							*F* (1, 38) =0.575
Northern America (RC)	5	23	0.11	0.816			
Europe	6	17	−0.03	−0.212	−0.14	−0.758	
Year of publication	11	41	−0.03	−0.364	−0.02	−1.515	*F* (1, 38) = 2.295
Impact factor of the journal	11	41	0.07	0.670	0.05	0.649	*F* (1, 38) = 0.421

*Note. k* = number of independent studies; #ES = number of effect sizes; *B*_0_/Cohen’s *d* = intercept; *t*_0_ = *t* value for testing the significance of the mean Cohen’s *d; B*_1_ = estimated regression coefficient (i.e., difference in Cohen’s *d* with reference category); *t*_1_ = test of regression coefficient; *F*(*df*_1_, *df*_2_) = omnibus *F* test; (RC) = reference category.

+ *p* < .10. *p < .05. **p < .01. ***p < .001.

## Discussion

This review presents the results of two meta-analyses on the effects of interventions that target moral development of youth engaged in delinquent behavior in order to reduce criminal offense recidivism. Although results show small-to-medium and significant effects of these interventions on moral judgment, no meta-analytic significant effects were found on criminal recidivism. We also found insufficient (quasi-) experimental studies to conduct meta-analyses of interventions that target empathy or self-conscious emotions (i.e., guilt and shame), which underlines the need for future research on this topic.

All interventions were group interventions focusing on moral judgment. Moderator analyses showed that ART/EQUIP did not have a significant effect on moral judgment, whereas other interventions yielded a significant and medium positive effect on moral judgment. An explanation for the non-significant ART/EQUIP effect may be found in a lack of program integrity, particularly in Europe (Helmond et al., 2015; Van Stam et al., 2014), which is in line with results from a meta-analysis by Goense et al. (2016), showing that only interventions carried out with high levels of program integrity produced positive outcomes in juveniles with antisocial behavior, including youth engaged in delinquent behavior. Other explanations could be that the effectiveness of ART/EQUIP, more than other interventions targeting moral development, depends on other treatments that youth engaged in delinquent behavior simultaneously receive or may have received in the past (Hoogsteder et al., 2014, 2021) or the quality of the living group climate in juvenile justice institutions (Stams & Van der Helm, 2017; Van der Helm et al., 2018), since EQUIP may be regarded as a just community intervention, which involves the social environment (Gibbs et al., 1995; Kohlberg et al., 1975).

Given the low statistical power in the moderator analyses that were performed in the current meta-analyses, it seems relevant to emphasize that the direction of some non-significant results of our meta-analyses is in line with findings from a previous meta-analysis by Stams et al. (2006), showing that production measures yielded larger effects than reproduction measures of moral judgment, as well as the studies by Weisburd et al. (2001) and Welsh et al. (2011), showing that RCTs yielded less favorable effects of judicial interventions than quasi-experimental designs. In addition, although non-significant, the results from the moderator analysis on type of offense were as expected, indicating that a delay in moral judgment was associated with minor and general offenses, but not with violent offenses.

Despite vast empirical evidence showing that moral development of youth engaged in delinquent behavior is associated with juvenile delinquency (Spruit et al., 2016; Stams et al., 2006; Van Langen et al., 2014; Van Vugt et al., 2011), with the strongest association for moral judgment (large effect), we found an almost zero and non-significant effect of interventions targeting moral development (i.e., moral judgment) of youth engaged in delinquent behavior on criminal recidivism. A first explanation is that a high proportion of youth engaged in delinquent behavior may be unsuitable for group treatment (Hoogsteder et al., 2015). Group interventions always carry the risk that young people with antisocial behavior will negatively influence each other, thus reinforcing each other’s deviant behavior (e.g., Dishion & Dodge, 2005; Poulin et al., 2001). However, ART and especially EQUIP both take steps to prevent such contamination.

The results of our meta-analytic review may indicate that targeting one component of moral development only, particularly moral judgment, is insufficient to reduce criminal offense recidivism, despite the fact that various interventions also targeted several relevant social and cognitive skills. The effects could have been different if treatment had also focused on moral emotions, because moral emotions have been shown to play a role in the translation of moral judgment into morally relevant behavior (Chapman et al., 2009; Pizarro, 2000), and because most juveniles who show delinquent behavior not only display deficiencies in moral judgment, but also in empathy, shame, and guilt (Schalkwijk et al., 2016).

The general lack of effects on criminal recidivism of interventions that target moral development may be explained by the narrow focus of most of these interventions, except EQUIP, which may insufficiently take into account the multi-causal determination of delinquency. Criminal behavior is determined by multiple criminogenic factors, and not by moral development alone (Andrews et al., 2008). For instance, many juvenile offenders have a history of externalizing-inducing traumatic life events, which may make them vulnerable for aggressive and delinquent behavior (Asscher et al., 2014). Without taking into account factors that cause or contribute to delinquency, such as (traumatic) stress, it may not be possible to effectively treat aggressive and delinquent behavior among youth (Hoogsteder et al., 2022; Somers et al., 2024). It may therefore be necessary to first ensure that youth engaged in delinquent behavior can acquire adequate self-regulation skills, including impulse control and emotion regulation (Hoogsteder et al., 2018), while cognitive biases related to aggressive and delinquent behavior are reduced, as for example in EQUIP (Helmond et al., 2014; Hoogsteder et al., 2014). This will probably require multimodal interventions, in which the focus is placed on several criminogenic factors simultaneously (Andrews & Bonta, 2006, 2010), maximizing responsiveness to the delinquent’s criminogenic needs through personalization of treatment (Hoogsteder et al., 2015).

The present meta-analytic study has some limitations. A first limitation concerned insufficient statistical power to detect small or small-to-medium differences in moderator analyses. A second limitation was that many studies did not report basic participant or study characteristics, which precludes the examination of comprehensive moderator analyses with sufficient statistical power. For instance, only two studies examined female delinquents, but did not report separate effect sizes for male and female delinquents. In addition, we could not distinguish between different ethnicities due to lack of detailed information on cultural background of juvenile offenders. We therefore chose to code percentage of White participants, which unfortunately does not adequately represent the multi-cultural diversity in both US and Europe. A third limitation was that studies did not assess treatment integrity, which is seen as a prerequisite for robust research on the effectiveness of any behavioral or social (judicial) intervention. Finally, as long as clinical trials are not registered before the start of effectiveness research, it remains difficult to adequately account for publication bias. Even robust trim-and-fill analysis cannot solve this problem. We therefore cannot rule out the possibility of publication bias, although we found no indications of publication bias in neither of the two meta-analyses.

## Conclusion

The systematic search performed in the current review revealed that, although moral development interventions are frequently applied in the field, there are relatively few controlled evaluation studies of these interventions. Additionally, treatment integrity or level of program implementation was not assessed in any of these studies. Nevertheless, it was possible to perform a first meta-analysis of the effectiveness of moral development interventions that focused on moral judgement. There is a strong need for research to further evaluate and develop interventions that focus on moral development in youth engaged in delinquent behavior, taking into account treatment integrity and level of program implementation, which should be assessed with valid and reliable instruments (see Goense et al., 2016; Lipsey et al., 2001). Notably, group interventions may not sufficiently contribute to individual aspects of underlying problems and changes in moral behavior. Therefore, the question emerges how to ensure that moral interventions work? We suggest that interventions should target self-regulation skills (reducing stress and impulsivity and improving emotion regulation), psychological problems (e.g., traumatic stress) that are associated with aggression and delinquent behavior, and multiple aspects of moral development (self-conscious emotions, empathy, and moral judgment) in tailor-made treatment, which takes into account individual characteristics of offenders, including their level of cognitive functioning, treatment motivation, cultural background, and social environment (Andrews & Bonta, 2010).

## Figures and Tables

**Appendix 3.**
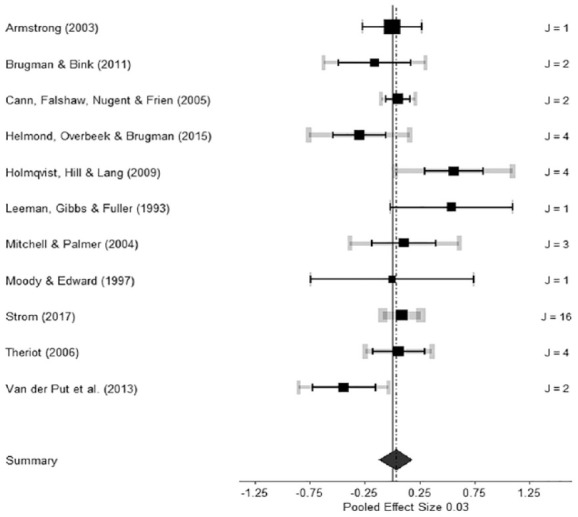
Forest Plot of the Effects of Moral Development Interventions on Criminal Recidivism. *Note*. A rectangle representing the meta-analytic study mean has been depicted in each line along with its 95% CI in bold black; the grey CI is based on the sampling variance of individual observed ESs of the study. The thickness of the grey confidence intervals is proportional to the number of effect sizes reported within studies (Fernández-Castilla et al., 2020). J = number of effect sizes.

**Appendix 4.**
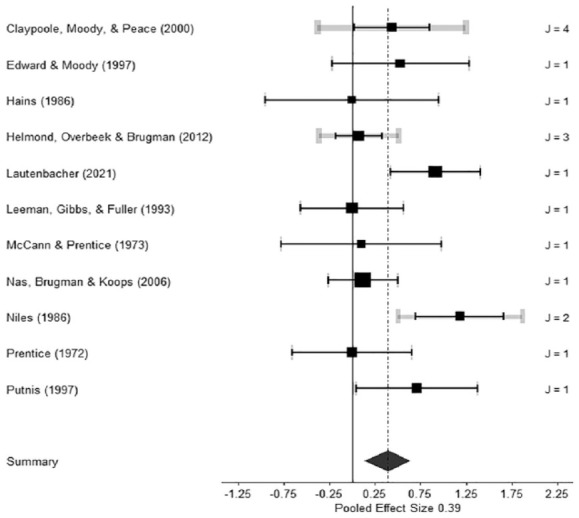
Forest Plot of the Effect of Moral Development Interventions on Moral Judgment. *Note*. A rectangle representing the meta-analytic study mean has been depicted in each line along with its 95% CI in bold black; the grey CI is based on the sampling variance of individual observed ESs of the study. The thickness of the grey confidence intervals is proportional to the number of effect sizes reported within studies (Fernández-Castilla et al., 2020). J = number of effect sizes.

## Appendix

**Appendix 1.** Characteristics of Primary Studies Moral Judgment.

No.	Author (year)	*# ES*	*N*	Continent	Intervention	Study design	Control group	Outcome	% caucasian	Gender	Mean age	Type delinquency
1	Nas et al. (2006)	1	108	EU	EQUIP	Quasi-experimental	NT	Moral judgment		Male	16.83	Violent
2	Helmond et al. (2012)	3	115	EU	EQUIP	Quasi-experimental	TAU	Moral judgment	39	Mixed	15	General
3	Leeman et al. (1993)	1	57	USA	EQUIP	RCT	NT	Moral judgment	66.67	Male	16	Minor
4	Claypoole et al. (2000)	4	48	USA	EQUIP Oriented	Quasi-experimental	TAU	Moral judgment	42	Both	15.10	General
5	Niles (1986)	2	17	USA	Moral Discussion Group	Quasi-experimental	Placebo & NT	Moral judgment		Male	14.50	Minor
6	Putnins (1997)	1	38	AUS	Other	Quasi-experimental	NT	Moral judgemment		Mixed	16.70	General
7	Prentice (1972)	1	36	USA	Other	RCT	Placebo	Moral judgment		Male	13.00	General
8	McCann and Prentice (1973)	1	20	USA	Other	RCT	NT	Moral judgment	5	Male	12.60	Minor
9	Moody (1997)		28	USA	Other	Quasi-experimental	TAU	Moral judgment	10.71	Male	14.25	Minor
10	Hains et al. (1986)	1	17	USA	Moral Discussion Group	Quasi-experimental	NT	Moral judgment	65	Male	17	Sexual
11	Lautenbacher et al. (2021)	1	75	EU	Other	Quasi-experimental	NT	Moral judgment		Male	15.25	Minor

*Note.* No. = study number; #ES = number of effect sizes computed from primary study; *N* = number of participants; Continent = location of study, EU = Europe, N-A = North America; Intervention = type of intervention used in primary Study, R&R = Reasoning and Rehabilitation, ART = Aggression Replacemnt Training; Study design = type of study design used in primary study, RCT = Randomized Controlled Trial; Control group = type of control group in primary study, NT = No Treatment, TAU = Treatment As Usual; Intention to treat, Yes = starters, No = completers, Both = the primary study reported an effect size with starters and completers; Outcome = type of outcome,; % caucasian = percentage caucasian participants in primary study.

**Appendix 2.** Characteristics of Primary Studies Recidivism.

No.	Author (year)	#ES	*N*	Continent	Intervention	Study design	Control group	Outcome	% caucasian	Gender	Mean age	Type delinquency
1	Mitchell and Palmer (2004)	3	62	EU	R&R	Quasi-experimental	NT	Both	87	Male	17.65	General
2	Moody (1997)	1	28	N-A	Other	Quasi-experimental	TAU	Reoffense	21	Male	14.25	General
3	Van der Put et al. (2013)	2	192	EU	ART/EQUIP	Quasi-experimental	TAU	Reoffense	63	Mixed	16.70	General, violent
4	Cann et al. (2005)	2	1,735	EU	R&R	Quasi-experimental	NT	Reoffense	-	Male	20.40	General
5	Armstrong (2003)	1	212	N-A	Other	RCT	NT	Reoffense	32	Male	21.45	General
6	Brugman and Bink (2011)	2	77	EU	ART/EQUIP	Quasi-experimental	TAU	Both	-	Male	17.23	General
7	Helmond et al. (2015)	4	132	EU	ART/EQUIP	Quasi-experimental	TAU	Reoffense	41	Mixed	15.70	General
8	Leeman et al. (1993)	1	57	USA	EQUIP	RCT	TAU	Reoffense	66.7	Male	16.00	General
9	Theriot (2006)	4	175	N-A	Other	Quasi-experimental	NT	Both	82.4	Mixed	15.85	Drug related
10	Holmqvist et al. (2009)	4	57	EU	ART	Quasi-experimental	TAU	Reoffense	-	-	17.17	General
11	Strom et al. (2017)	16	258	USA	Other	Quasi-experimental	NT	Police contact	0.18	Mixed	14.40	Minor, violent, drug related

*Note*. No. = study number; #ES = number of effect sizes computed from primary study; *N* = number of participants; Continent = location of study, EU = Europe, N-A = North America; Intervention = type of intervention used in primary study, R&R = Reasoning and Rehabilitation, ART = Aggression Replacemnt Training; Study design = type of study design used in primary study, RCT = Randomized Controlled Trial; Control group = type of control group in primary study, NT = No Treatment, TAU = Treatment As Usual; Intention to treat, Yes = starters, No = completers, Both = primary study reported an effect size of reoffense and time to reoffense; % caucasian = percentage caucasian participants in primary study.
